# Insights into the regulation of energy metabolism during the seed-to-seedling transition in marine angiosperm *Zostera marina* L.: Integrated metabolomic and transcriptomic analysis

**DOI:** 10.3389/fpls.2023.1130292

**Published:** 2023-03-10

**Authors:** Meiling Zhu, Yu Zang, Xuelei Zhang, Shuai Shang, Song Xue, Jun Chen, Xuexi Tang

**Affiliations:** ^1^ College of Marine Life Sciences, Ocean University of China, Qingdao, Shandong, China; ^2^ Key Laboratory of Marine Eco-Environmental Science and Technology, First Institute of Oceanography, Ministry of Natural Resources, Qingdao, Shandong, China; ^3^ College of Biological and Environmental Engineering, Binzhou University, Binzhou, Shandong, China

**Keywords:** seagrass, seed development, starch and sucrose metabolism, glycolysis, TCA cycle energy metabolism during seed development

## Abstract

Seed development is a crucial phase in the life cycle of seed-propagated plants. As the only group of angiosperms that evolved from terrestrial plants to complete their life cycle submerged in marine environments, the mechanisms underlying seed development in seagrasses are still largely unknown. In the present study, we attempted to combine transcriptomic, metabolomic, and physiological data to comprehensively analyze the molecular mechanism that regulates energy metabolism in *Zostera marina* seeds at the four major developmental stages. Our results demonstrated that seed metabolism was reprogrammed with significant alteration of starch and sucrose metabolism, glycolysis, the tricarboxylic acid cycle (TCA cycle), and the pentose phosphate pathway during the transition from seed formation to seedling establishment. The interconversion of starch and sugar provided energy storage substances in mature seeds and further acted as energy sources to support seed germination and seedling growth. The glycolysis pathway was active during *Z. marina* germination and seedling establishment, which provided pyruvate for TCA cycle by decomposing soluble sugar. Notably, the biological processes of glycolysis were severely inhibited during *Z*. *marina* seed maturation may have a positive effect on seed germination, maintaining a low level of metabolic activity during seed maturation to preserve seed viability. Increased acetyl-CoA and ATP contents were accompanied with the higher TCA cycle activity during seed germination and seedling establishment, indicating that the accumulations of precursor and intermediates metabolite that can strengthen the TCA cycle and facilitate energy supply for *Z*. *marina* seed germination and seedling growth. The large amount of oxidatively generated sugar phosphate promotes fructose 1,6-bisphosphate synthesis to feed back to glycolysis during seed germination, indicating that the pentose phosphate pathway not only provides energy for germination, but also complements the glycolytic pathway. Collectively, our findings suggest these energy metabolism pathways cooperate with each other in the process of seed transformation from maturity to seedling establishment, transforming seed from storage tissue to highly active metabolic tissue to meet the energy requirement seed development. These findings provide insights into the roles of the energy metabolism pathway in the complete developmental process of *Z*. *marina* seeds from different perspectives, which could facilitate habitat restoration of *Z*. *marina* meadows *via* seeds.

## 1 Introduction

Seagrasses are the only flowering plants (angiosperms) adapted to grow and reproduce entirely submerged in seawater (Tol et al., 2017). Similar to land angiosperms, seagrasses can produce seeds by reproducing sexually, which is a critical stage in the life cycle of seed-propagated plants (Tarquinio et al., 2021). *Zostera marina* is an important foundation species for coastal ecosystems in the northern hemisphere that provides essential ecosystem functions, including stabilizing sediments, providing nursery grounds for juvenile fish, and sequestering carbon (Fourqurean et al., 2012; Bengtsson et al., 2017). Despite their ecological importance, *Z*. *marina* meadows are degrading at an breakneck rate under anthropogenic impacts and natural threats (Orth et al., 2006; Waycott et al., 2009; Short et al., 2011). Thus, effective protection and active restoration engineering are becoming increasingly important. Seed restoration is the main method to repair damaged seagrass meadows because it has a low cost, maintains population genetic diversity, and causes less harm to the original seagrass meadow (Yue et al., 2019). However, such restoration is limited by the low germination rate of seedlings and poor seedling establishment after seed germination (Orth et al., 2010). Therefore, determining the changes in key physiological processes during seed development for seagrasses is important for promoting the successful regeneration of seagrass meadows.

Seed maturation, germination, and seedling establishment are vital stages during a plant’s life cycle (Ma et al., 2017). These stages are precisely regulated by various biological processes involving signaling transduction, phytohormone regulation, and energy metabolism (Hao et al., 2022). Previous studies have shown that energy metabolism plays a dominant role in regulating seed development; it provides the material and energy basis for seed development by regulating the synthesis, hydrolysis, and conversion of storage compounds, such as starch, storage proteins, and lipids (Liu et al., 2015; Ali and Elozeiri, 2017; Ma et al., 2017). Starch is the major form of carbohydrates and energy carriers in plant storage organs (Ma et al., 2020). The amylolytic breakdown of stored starch in seeds during germination is an important biochemical event that provides reducing sugars as energy sources to support the early stages of seed germination in angiosperm plants (Chen et al., 2019). Seed germination and seedling establishment are accompanied by intense and complex physiological metabolic activity (Zienkiewicz et al., 2014). Most storage substances begin to be mobilized, and three respiratory pathways namely glycolysis, the pentose phosphate pathway, and the tricarboxylic acid cycle (TCA cycle) are activated to provide materials and energy for subsequent seedling growth and development (Song et al., 2021). For example, glycolysis provides basic intermediates and energy upon seed germination, and the TCA cycle is crucial for energy generation during seed germination by supplying reducing equivalents for the operation of the respiratory chain (Tan et al., 2013). Moreover, the pentose phosphate pathway can provide not only pentose phosphate for nucleotide metabolism, but also NADH for different biosynthesis reactions to promote seed germination and seedling growth (He et al., 2011). However, some studies have indicated that changes in the enzymes involved in the TCA cycle are not detected during the germination of cereal seeds, and the energy demand seems to be fulfilled mainly by glycolysis (Yang et al., 2007). In addition, other studies have demonstrated that glycolysis and the TCA cycle provide most of the energy for seed germination, while the pentose phosphate pathway plays a small contributory role in some angiosperm seed development (Han et al., 2014; He et al., 2019).

Although the number of studies about the complex series of physiological changes in terrestrial plants during seed development is gradually increasing (Yan and Chen, 2017; Lee et al., 2018), physiological responses and molecular regulatory mechanisms in the process of seed development for marine angiosperms still need to be further explored (Sugiura et al., 2009). In particular, the physiological changes in the complete seed development process have not been studied rigorously. To gain a further understanding of the role and regulation of energy metabolic characteristics during seed maturation and germination in *Z*. *marina*, we analyzed the morphological and physiological characteristics, as well as the changes in transcription and metabolism at four different stages, from seed maturation to seedling establishment. This study provides novel insights into the roles of the energy metabolism pathway during the crucial developmental phase in *Z*. *marina* seeds and facilitates its future application in promoting seed germination to improve seagrass conservation effectiveness.

## 2 Materials and methods

### 2.1 Plant materials and growth conditions

*Zostera marina* seeds were randomly collected from May to September 2021 from a shallow seagrass meadow in Changdao County, Yantai City, China. To avoid potential bias in seed vigor due to aging, only the seeds produced from the current year were used in this study. Immature seeds were distinguished from those of the different developmental stages by selecting soft and green seeds that were still attached to spathes at the time of collection. Mature reproductive shoots were picked and placed in a 600-μm mesh bag, which was suspended in seawater at sampling sites for 1 month until mature seeds were released from the rotten shoots. Mature seeds were sieved (2-mm) from the decaying shoots and brought back to the laboratory in natural seawater. The mature seeds were rinsed repeatedly with seawater and then planted in a large box containing sieved sediment at 2–3 cm deep. The box was placed in aerated flow-through tanks filled with natural seawater at a salinity of 30 ± 0.2 psu and a temperature of 15°C. The seawater was changed weekly to observe the germination of *Z. marina* seeds.

Yellow seeds with soft in texture and neatly arranged in the spathe were chosen as immature stage (stage I). Black or brown seeds with a very firm and hard texture were defined as mature stage (stage II). The cotyledons broke through the seed coat was considered as germinated stage (stage III). Seeds that grew their first young leaves in aquariums were marked as the seedling establishment stage (stage IV). Healthy seeds were collected at different stages, including stage I, II, III, and IV, and were frozen in liquid nitrogen for further analysis.

### 2.2 Morphological and histological observations

Light microscope was prepared for observing the morphological alterations of *Z. marina* seeds at differernt stage. For histological procedures, three biological replications (cotyledon of three seeds) were performed for periodic acid–schiff (PAS) stainning at each developmental stage. Samples were fixed by FAA and embedded by paraffin to make paraffin sections with 6–10µm slices using a LEICA2150 rotary microtome (Leica, Germany). After dewaxing, the slices were stained in PAS solution for the observation of starch changes using an Olympus BX-61 microscope (Olympus, Japan).

### 2.3 Physiochemical analysis

The 0.1g frozen seeds/seedlings from each repetition were weighed and ground into fine powder in liquid nitrogen for subsequent indicator determination. The starch content was quantified using the anthrone sulfuric acid method (Wang et al., 2016b). The starch was decomposed into glucose by acid hydrolysis and then reacted with anthrone, that has the absorption peak at a wavelength of 620 nm. Sucrose and fructose contents were tested by resorcinol and colorimetric methods (Du et al., 2020). The product of the reaction of fructose and resorcinol has a maximum absorption value at 480 nm and further calculated the content of sucrose, which could be hydrolyzed to fructose under acidic conditions. The glucose content was measured according to the glucose oxidase/peroxidase method (Kabasakalian et al., 1974). After 0.1g frozen seed samples was homogenated with 1 mL of distilled water and extracted at 100°C for 10min, the supernatant was used to determining the content of glucose after centrifugation at 8000 g at 25°C for 10 min by glucose detection kit (Solarbio, Beijing, China). For measuring the amino acids (AAs) content, the ninhydrin assay method was used (Lee et al., 2003). α-amino group with ninhydrin results in bluish violet compounds with a characteristic absorption peak at 570 nm. Pyruvic acid, total proteins, and ATP content by pyruvate content assay kit, BCA protein kit and ATP content assay kit obtained from Solarbio Company (Beijing, China). The free fatty acid (FFA) content was using a FFA assay kit (Comin Biotechnology Co. Ltd, Suzhou, China).

Frozen samples weighing 0.1 g were ground with liquid nitrogen to make tissue homogenates for enzyme activity analysis. The activities of α-amylase and ADP-glucose pyrophosphorylase (AGPase) were conducted using amylase activity assay kit (Comin) and AGP activity assay kit (Comin). The seeds for each stage were measured across three biological replicates, where each biological replicate included three technical replicates.

### 2.4 Transcriptome analysis and data processing

Trizol reagent was used to extract total RNA from each stage. Four biological repetitions were performed for each stage, and each repetition contained 200 mg of *Z*. *marina* seeds. The 2100 Bioanalyzer (Agilent) was used to assess RNA quality, and RNA quantification was confirmed by ND-2000 (NanoDrop Technologies). The cDNA library construction was processed using a TruSeq™ RNA sample preparation kit (Illumina, SanDiego, Canada) and was sequenced with an Illumina NovaSeq 6000 sequencer (2×150 bp read length).

Clean paired-end reads were aligned against the *Z*. *marina* reference genome (*Z*. *marina*_668_v3.1) using HISAT2 (http://ccb.jhu.edu/software/hisat2/index.shtml, version 2.1.0) to get mapped reads, and gene abundances were quantified *via* RSEM (http://deweylab.biostat.wisc.edu/rsem/, version 1.3.3) to calculate FPKM values. We annotated the DEGs with the genome annotation file “*Z. marina*_668_v3.1.annotation_info.txt” from the Joint Genome Institute (JGI) database. The DEGs between any two adjacent developmental stages were screen out using DESeq2 (http://bioconductor.org/packages/stats/bioc/DEScq2, version 1.10.1) with |log_2_FC| > 1 and FDR <0.05 based on FPKM rasults. The biological function of these DEGs was further determined by the KEGG enrichment analysis (http://www.genome.jp/kegg/, version 2017.08), and a *P*-value ≤ 0.05 was considered significant enrichment.

### 2.5 Metabolite extraction and LC-MS/MS analysis

To further measure the change in energy metabolites, we performed liquid chromatography-tandem mass spectrometry (LC-MS/MS) analysis. *Z*. *marina* seeds from the four stages were harvested with four independent biological replicates. A total of 50 mg of each repetition was used for the extraction of metabolites with 500 μL precooled methanol at -20°C. Then the mixture was homogenized by vortexing for 3min and centrifuged for 10 min (12,000 g, 4°C) to collect the supernatant. The loading solution was obtained by passing 200 μL of the supernatant over the protein pellet plate for further analysis by Q-Trap 6500+ mass spectrometer linked to UPLC system. The ACQUITY UPLC BEH Amide column (1.7 µm, 100 mm×2.1 mm i.d.) was used (column temperature: 40°C, injection volume: 2 μL). Ultra-pure water (phase A) + 90% acetonitrile (phase B) were used to constitute the mobile phase, which was run at 0.40 mL/min.

Based on the standard-built database MWDB, the mass spectrometry detection data were qualitatively analyzed, and a quantitative analysis was performed in MRM mode. Then, significantly differential metabolites between groups were filtered under the condition of VIP value ≥ 1 and *P*-value ≤ 0.05.

### 2.6 Statistical analysis

Heatmap and clustergram analysis (“heatmap” R package, version 1.0) for four replicates were performed on the log 10 (FPKM) values of genes. Statistical analyses of physiological indicators and metabolite content were analyzed using SPSS 25.0. Measurement data were presented as means ± SD for three replicates. All data were first tested for homogeneity of variance using Levene’s tests and for normality using K-S test. The differences between adjacent stages were compared by a one-way analysis of variance (ANOVA), followed by the SNK test to evaluate statistical significance. Significance levels for all tests were set at a *P*-value < 0.05.

## 3 Results

### 3.1 Morphologic and cellular structure changes of seeds

**Figure 1:**
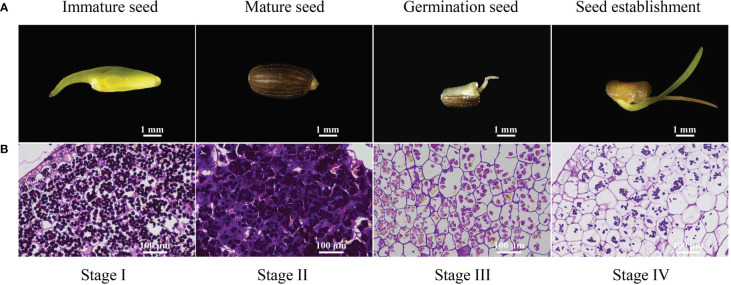
Morphology and histology of developing *Zostera marina* seeds. **(A)** *Zostera marina* seed at different developmental stages. **(B)** During *Zostera marina* seed development, starch was stained with PAS. The rulers indicate 1 mm in **(A)** and 200 μm in **(B)**.

In this experiment, we observed the morphological changes in the complete *Z*. *marina* seeds development process to better understand the characteristics of physiological metabolism changes during seed maturity, germination, and seedling establishment (**Figure 1A**). The seeds were neatly arranged in spathes, yellow-green and soft in texture at the immature seed stage. Mature seeds were released from spathes, had a hard texture, and were brown or black. Seed germination began with cracking in the seed coat and incomplete embryo exposure. The germ embryo of germinated seeds further developed and differentiated into one young leaf during the seedling establishment process.

The changes in starch granules in the cotyledons during the four stages were observed under a microscope after the adoption of PAS. The number of starch grains increased significantly from stage I to II, while the number of starch grains consistently decreased from stage II to IV (**Figure 1B**). This indicated that the starch content changed dramatically from seed maturity to seedling establishment.

### 3.2 Physicochemical index changes in the energy metabolism of seeds

**Figure 2:**
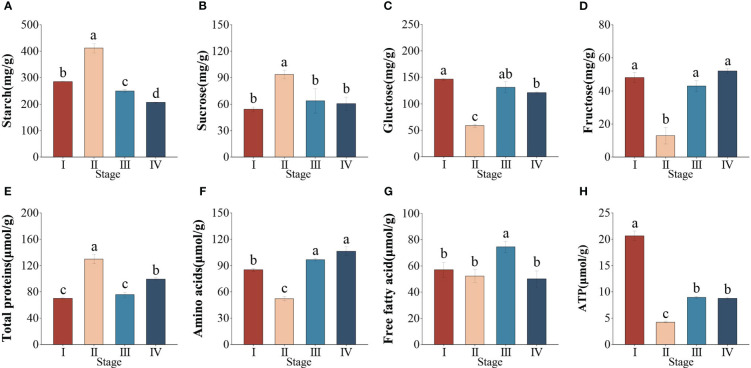
Changes in the content of energy metabolism metablites during different developmental stages in *Zostera marina* seeds. **(A)** Starch, **(B)** sucrose, **(C)** glucose, **(D)** fructose, **(E)** total proteins, **(F)** amino acids, **(G)** free fatty acids, **(H)** ATP. Values are the means ± standard deviation (n = 3). Different letters represent significant differences (*P* < 0.05).

To better understand changes in energy metabolism during the different seed developmental stages, we further measured the content of related metabolites. The sucrose, starch, and total proteins contents remarkably increased by 40, 31 and 46%, respectively from stage I to II (*P* < 0.05). However, after seed maturation, the starch, sucrose, and total protein contents were reduced by 59, 36, and 23%, respectively (**Figures 2A, B, E**). Both glucose and fructose contents decreased significantly at stages I–II (*P* < 0.05), followed by a marked increase at stages II–III (**Figures 2C, D**). In addition, the amino acids (AAs) and free fatty acid (FFA) contents decreased from stage I to II, while the contents increased from stage II to IV (**Figures 2F, G**). The ATP content markedly decreased from stage I to II but increased significantly from stage II to IV, the maximum occurred at stage I (**Figure 2H**). This suggested that changes in energy metabolism occurred in different seed developmental stages.

Significant changes in enzyme activity were found in *Z*. *marina* seeds at different developmental stages (*P* < 0.05). AGPase activity was markedly increased from stage I to II and then sustainably decreased, with the minimum occurring at stage IV (**Supplementary Figure 1A**). In addition, the activity of α-amylase was not significantly changed from stage I to II and then slightly rose in stage III, followed by a marked increase at stage IV (**Supplementary Figure 1B**).

### 3.3 Global analysis of dynamic changes during *Z*. *marina* seed development by transcriptomic and metabolic profiling

**Figure 3:**
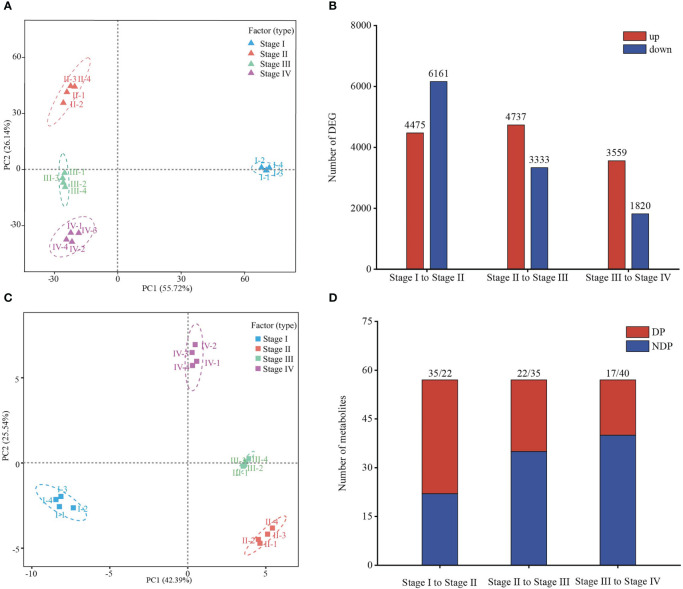
Transcriptomic and metabolic analyses reveal a dynamic transition during *Zostera marina* seed development. **(A, C)** PCA of transcriptome and metabolome during *Zostera marina* seed development. **(B)** Numbers of up- and down- regulated genes at different stages. **(D)** Number of differential metabolites during the transition between development stages. DP, differential metabolites. NDP, non-differential metabolites.

To identify key genes involved in regulating *Z*. *marina* seed maturity, germination, and transition to seedling establishment, we first carried out RNA-seq analysis in samples from four stages. A total of 121.16 Gb of clean sequencing data from 16 samples were produced, and the average clean data from each sample were more than 6.69 Gb. The rate of alignment between sequencing reads and the reference genome fell in the range of 82.51–93.88%, the GC content (44.1–46.77%) and high Q30 value (94.58–95.57%) enabled the subsequent differential expression analysis (**Supplementary Table 1**). Principal component analysis (PCA) showed significant differences among the four different stages and good intra‐group repeatability (**Figure 3A**). Differentially expressed genes (DEGs) were screened based on two aspects: log2 (fold change) and the statistical significance level. Pairwise differential expression profiling analysis was used to count the number of up- or down-regulated DEGs between any two neighboring stages. There were 14390 DEGs identified based on pairwise comparison analysis, inculding 10636 (4475 up- and 6161 downregulated) in stage I to stage II, 8070 (4737 up- and 3333 downregulated) in stage II to stage III and 5379 (3559 up- and 1820 downregulated) DEGs were screened out between stage III and stage IV, indicating a significant difference in different stages (**Figure 3B**, **Supplementary Table 2**). And a Venn diagram showedthe stage-specific expression genes between two neighboring stages, indicated the each stage specificity (**Supplementary Figure 2**).

To better explore the dynamics of energy metabolism from *Z*. *marina* seed mature to seedling establishment at the metabolic level, we performed targeted metabolomic profiling analysis for research in energy metabolomics using a liquid chromatography-tandem mass spectrometry (LC-MS/MS) platform. 57 metabolites related to energy metabolism in *Z*. *marina* seeds of four stage were detected. PCA revealed that different stages formed a significant separate cluster, indicating that metabolic changes occurred in different seed developmental stages (**Figure 3C**). Various numbers of significant metabolites among neighboring stages were identified. Similar to differential gene expression analysis, the number of significantly differential metabolites was relatively large between stage I and stage II (35 differential metabolites), followed by 22 the differential metabolites were identified in stage II to stage III, and 17 the differential metabolites in stage III to stage IV, providing important information combined with transcriptome data for the further exploration of energy metabolism dynamics (**Figure 3D**, **Supplementary Table 3**).

### 3.4 KEGG enrichment analysis of DEGs during *Z*. *marina* seed development

**Figure 4:**
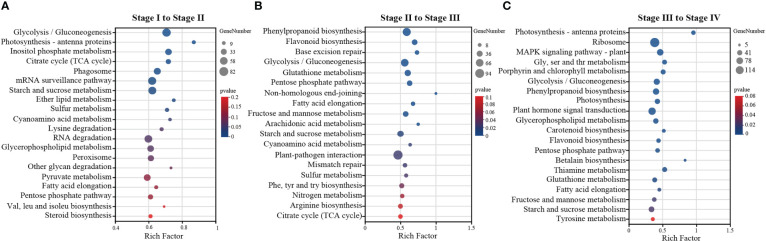
KEGG enrichment analysis of DEGs identified by pairwise comparison were performed by R package (version 1.0) clusterProfiler. **(A)** stage I to stage II, **(B)** stage II to stage III, **(C)** stage III to stage IV. The y-axis is the name of KEGG pathway, x-axis represents the ratio of the number of genes annotated in this pathway to background differentially expressed genes in this pathway. Size of circles indicates the number of enriched genes in each pathway, and the color represents the level of significance.

To understand the most involved metabolic pathways during *Z*. *marina* seed development, we tried to map the processes by functionally annotating the DEGs against the KEGG database. KEGG enrichment analysis found that from stage I to II (**Figure 4A**), glycolysis/gluconeogenesis was significantly enriched, following by photosynthesis-antenna proteins, inositol phosphate metabolism and citrate cycle (TCA cycle) (p<0.01). In addition, the number of DEGs was also very high in starch and sucrose metabolism (p<0.05). There were ten extremely significant enrichment pathways from stage II to III (p<0.01): such as glycolysis/gluconeogenesis, pentose phosphate pathway and fructose and mannose metabolism. Followed by the starh and sucrose metabolism also was a significant enrichment pathway (p<0.05) (**Figure 4B**). As shown in **Figure 4C**, there are 19 significantly enriched metabolic pathways from stage III to IV (p<0.05), such as the largest KEGG term was “ribosome” with 114 genes. Additionally, 48 genes were associated with the “glycolysis/gluconeogenesis pathway,” 40 with “starch and sucrose metabolism,” and 53 with “MAPK signaling pathway-plant.” KEGG analysis showed that pathways related to energy metabolism dominated, such as starch and sucrose metabolism, glycolysis, the TCA cycle, and the pentose phosphate pathway.

### 3.5 Dynamic regulation of key genes and metabolites in starch and sucrose metabolism during seed development

**Figure 5:**
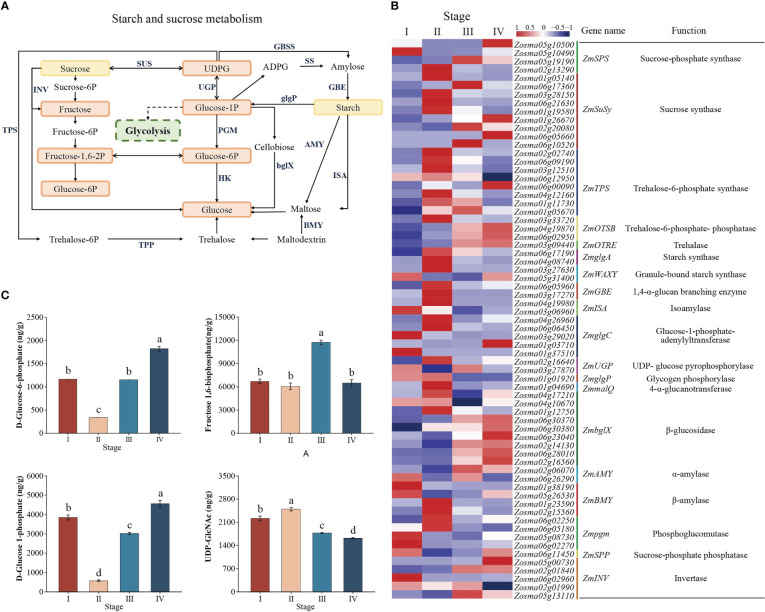
Starch and sucrose metabolism pathway. **(A)** Model and key components of starch and sucrose metabolism in *Zostera marina* seeds. The highlighted boxes indicate the metabolites quantified from the LC–MS/MS results. **(B)** Heatmap analysis of DEGs (log 10 (FPKM) values) involved in starch and sucrose metabolism. **(C)** Analysis of changes in the metabolites represented in starch and sucrose metabolism during *Zostera marina* seed development.

To identify important genes regulating energy metabolism during *Z*. *marina* seed developmental processes, we searched the major components in the starch and sucrose metabolism pathway based on KEGG enrichment analysis (**Figure 5A**). A total of nine genes were annotated as *ZmSuSy* encoding sucrose synthase (SUS); six were upregulated at stage II, and five were upregulated at stage III, suggesting that these genes might play differential roles in sucrose synthesis and degradation. The *ZmUGP*, *ZmSS*, and *ZmAGP* genes encoding UDP-glucose pyrophosphorylase (UGPase), starch synthase (SS), and AGPase, respectively, had a high expression level in stages I and II, while the expression levels of *ZmSS* and *ZmAGP* were significantly downregulated after stage II. In addition, the *ZmAMY*, *ZmbglX*, and *ZmINV* genes encoding α-amylase (AMY), β-glucosidase (bglX), and invertase (INV), respectively, were overall upregulated after stage II (**Figure 5B**, **Supplementary Table 4**). However, at the metabolome level, the glucose 1-phosphate (G1P), glucose 6-phosphate (G6P) and fructose 1,6-bisphosphate (FDP) content decreased, while the UDP-glucose (UDPG) and starch content increased from stage I to II. However, the G1P, G6P and FDP content increased, but UDPG content decreased from stage II to IV (**Figure 5C**). It was possible that more G1P was used to synthesize UDPG and thus starch.

### 3.6 Dynamic regulation of key genes and metabolites in glycolysis and the pentose phosphate pathway during seed development

**Figure 6:**
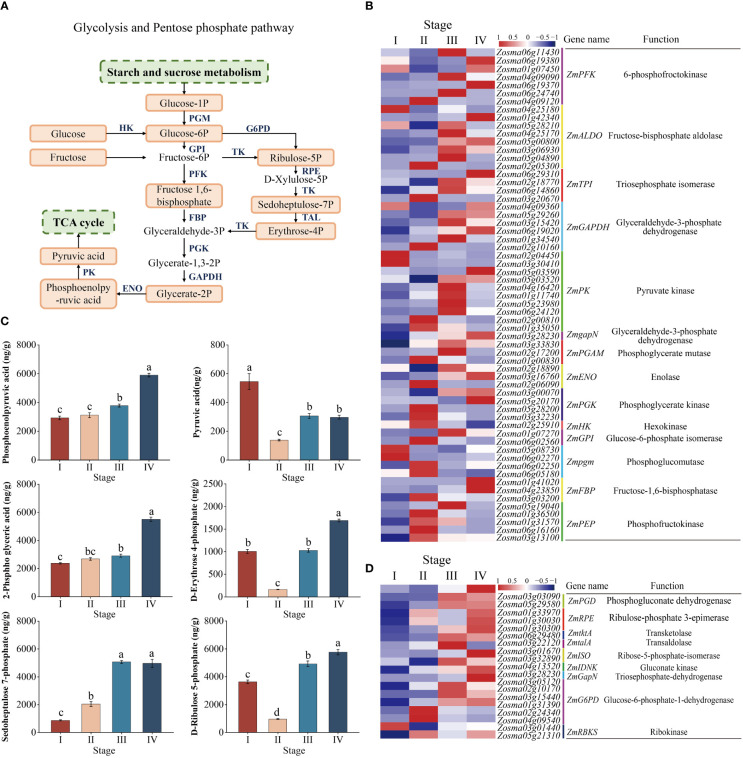
Glycolysis pathway and pentose phosphate pathway. **(A)** Model and key components of the glycolysis pathway and the pentose phosphate pathway in *Zostera marina* seeds. The highlighted boxes indicate the metabolites quantified from the LC–MS/MS results. **(B)** Heatmap analysis of DEGs (log 10 (FPKM) values) involved in the glycolysis pathway. **(C)** Analysis of changes in the metabolites represented in the glycolysis and pentose phosphate pathways during *Zostera marina* seed development. **(D)** Heatmap analysis of DEGs involved in the pentose and phosphate pathways.

KEGG analysis showed that glycolysis and pentose phosphate processes were significantly enriched in the seed maturation to seedling establishment and the relevant components in the pathway were drawn (**Figure 6A**). To better verify the molecular regulation of energy metabolism during seed development, we examined the DEGs involved in multiple processes, including genes functioning in glycolysis and pentose phosphate (**Figures 6B, D**, **Supplementary Table 4**). In the glycolytic pathway, 67 DEGs were enriched. The expression of most transcripts (e.g., *ZmENO*, *ZmPFK*, *ZmGAPDH*, and *ZmPK*) was downregulated from stage I to II, while the expression of these genes was significantly upregulated from stage II to IV (**Figure 6B**). Glycolysis establishes links to the pentose phosphate pathway *via* G6P. The important components in the pentose phosphate pathway include *ZmtktA*, *ZmG6PD*, *ZmPGD*, and *ZmtalA*. The expression of these genes was continually upregulated from stage I to IV (**Figure 6D**).

The changes in the metabolome were roughly consistent with transcriptional changes. In glycolysis, the content of pyruvic acid decreased from stage I to II, while there was no significant change in the content of PEP and 2-Phospho-D-glyceric acid (2-PGA). However, the content of these metabolites significantly increased from stage II to IV (**Figure 6C**). In the pentose phosphate pathway, our metabolic profiling analysis showed that the content of erythrose 4-phosphate and ribulose 5-phosphate were decreased from stage I to II, followed by an increase from stage II to IV. In addition, the content of sedoheptulose 7-phosphate was continually increased from stage I to IV (**Figure 6C**).

### 3.7 Dynamic regulation of key genes and metabolites in the TCA cycle pathway during seed development

**Figure 7:**
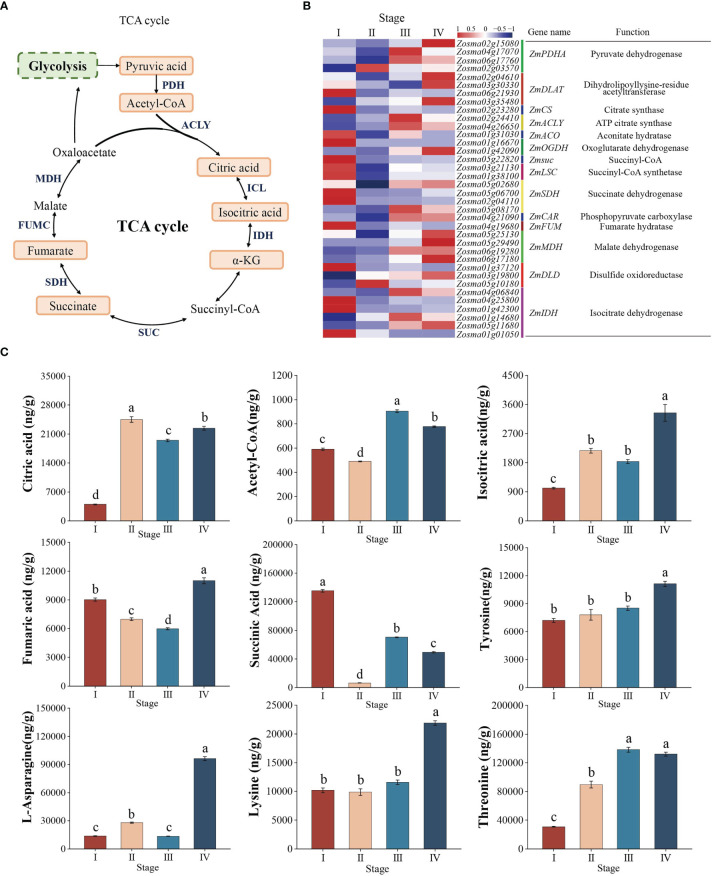
TCA cycle. **(A)** Model and key components of the TCA cycle in *Zostera marina* seeds. The highlighted boxes indicate the metabolites quantified from the LC–MS/MS results. **(B)** Heatmap analysis of DEGs (log 10 (FPKM) values) involved in the TCA cycle. **(C)** Analysis of changes in the represented metabolites in the TCA cycle during *Zostera marina* seed development.

According to the results of KEGG enrichment analysis, the TCA cycle pathway diagram was drawn (**Figure 7A**). A total of 37 DEGs were significantly enriched in this pathway in *Z*. *marina* seed development (**Figure 7B**, **Supplementary Table 4**). *ZmMDH*, *ZmPDHA*, *ZmFUMC*, and *ZmACO* were significantly downregulated from stage I to II, while the expression of these genes were continuously upregulated from stage II to IV (**Figure 7B**). The metabolome data were roughly consistent with the expression data, the content of succinic acid and fumaric acid decreased from stage I to II, while the content of citric acid and isocitric acid increased. However, from stage II to IV, in addition to the decrease in the citric acid content, the content of succinic acid, fumaric acid, and isocitric acid increased (**Figure 7C**).

In addition, most AAs are important source of substrates for the TCA cycle. Our results showed that the content of lysine (Lys), threonine (Thr), asparagine (Asn), and tyrosine (Tyr) remained higher from stage III to IV (**Figure 7C**). This means that different AAs are catabolized and enter the TCA cycle, thereby increasing flux toward the TCA cycle.

## 4 Discussion

### 4.1 Interconversion of starch and sugar plays an important role during *Z*. *marina* seed development

Starch and sucrose regulatory pathway is crucial in maintaining the dynamic balance of sugar substances and energy homeostasis in plant growth and development by regulating the interconversion of starch and sugar (Zhao et al., 2022). Some studies have suggested that sucrose serves as a substrate for the formation of storage reserves and as a main nutrient and energy source for plant development (Yu et al., 2016; Ma et al., 2020). In the metabolic pathways of *Nelumbo nucifera*, sucrose is mainly produced in leaves by photosynthesis, transported to seeds, and then accumulated as energy reserves for seed maturation (Wang et al., 2016a). In the early seed germination stages, sucrose is predominantly hydrolyzed to generate glucose and fructose to provide energy and precursors for seed germination (Silva-Sanchez et al., 2013; Wang et al., 2016a; Mala et al., 2021). In accordance with a previous study, sucrose was considered to be one of the major carbohydrate reserves in mature *Z. marina* seeds (Touchette and Burkholder, 2000). In this study, the content of sucrose significantly increased from stage I to II, indicating that sucrose produced by leaf photosynthesis may be transported to the seed during *Z*. *marina* seed maturation. In addition, the increase in sucrose content might further regulated starch synthesis. In contrast, the content of sucrose decreased as the content of glucose and fructose increased from stage II to IV, indicating that sucrose can be hydrolyzed into hexose and further provide energy for seed germination and growth.

Sucrose synthase (SUS) has reversible activity, where it catalyzes sucrose synthesis and degradation, but hydrolytic activity is suggested to play a leading role in guaranteeing a steady concentration of UDP-glucose (UDPG) and providing energy for plant growth (Zhang et al., 2016). However, our results showed that the expression of *ZmSuSy*s affected sucrose homeostasis. Nine genes were annotated as *ZmSuSy* encoding sucrose synthase (SUS); six of these genes were upregulated at stage II, and five *ZmSuSy*s were upregulated at stage III, suggesting that *ZmSuSy* might play differential roles in sucrose synthesis and degradation during different development stages. Invertase (INV) irreversibly cleaves sucrose into glucose and fructose and is very active in rapidly elongated meristems, as well as in rapidly developed young organs or tissues (Zhu et al., 2018). Here, the expression of *ZmINV* tended to increase at stages III and IV, which indicated that *ZmINV* acted as a regulator to provide glucose and fructose for seed germination and seedling establishment.

Starch acts as the major carbohydrate storage form in mature seeds for degradating to generating energy and metabolites during germination and seedling growth (Tang et al., 2017). In the present study, the starch content significantly increased together with the decreased content of glucose from stage I to II. These results revealed a higher starch synthesis metabolism in seed maturation, thus ensuring an energy reserve for germinating seedlings. These findings are similar to studies on the seed development process in *Posidonia oceanica*, abundant seed storage (starch) appears to be a characteristic, and should be favorable for seedling establishment in a marine environment (Belzunce et al., 2005). Previous studies on the development of *Glycine max* seeds showed that the decrease in hexose phosphates (G6P, G1P, and FDP) and the nucleotide sugar UDPG, which were precursors to carbohydrate production, was accompanied by the accumulation of starch in the mature seed (Kambhampati et al., 2021). Our study showed that G6P, G1P and FDP decreased first from stage I to II and then increased from stage II to IV, while the content of UDPG and starch showed the opposite trend. This suggests that starch–sugar interconversion occurs throughout *Z. marina* seed development.

Starch synthesis occurs through the activity of three major enzymes: AGPase, SS, and UGPase. This systematic interaction of multienzymes gradually leads to synthesized starch (Chopra et al., 2005; Smith and Zeeman, 2020). Studies have demonstrated that the activity of AGPase and UGPase is correlated with the starch content and that starch synthase activity increases with the accumulation of starch during the early development of mung bean seeds (Chopra et al., 2005). We identified six genes encoding the main enzymes of starch biosynthesis. *ZmAGP*s and *ZmSS*s were upregulated at stage II and the activity of AGPase increased, which enhanced the carbon flux into starch. β-glucosidase (bglX) and amylase are the key enzymes involved in the process of starch decomposition, affecting seed germination and seedling growth (Zhao et al., 2022). α-amylase is a major carbohydrase that catalyzes the hydrolysis of α-(1, 4) glycosidic linkages in starch, glycogen, and related polysaccharides (Huang et al., 2019). The enzymes bglX belong to the glycosylhydrolase family, which catalyze the hydrolysis of glycosidic bonds and hydrolyze compounds such as cellobiose into glucose to regulate plant growth and development (Huang et al., 2019). In this study, most *ZmbglX*s along with *ZmAMY*s were upregulated, and α-amyase activity was significantly increased from stage II to IV, thereby accelerating starch metabolism and generating glucose. The glucose content increased to the highest level at stage IV, indicating that the processes of starch consumption and glucose accumulation were active at this stage. The findings was similar to previous findings in other seagrass seeds. The starch stored in mature seagrass seeds (*Thalassia hemprichii* and *Posidonia* species: *P. australis*, *P. sinuosa* and *P.coriacea*) were presumably hydrolyzed to sugar and usedto nourish early seedling growth (Kuo and Mccomb, 1989; Kuo and Kirkman, 1990; Kuo et al., 1990; Kuo et al., 1991).

### 4.2 Glycolysis provides an important pivotal substance for the seed germination process

Glycolysis is a main catabolic pathway of glucose metabolism and can provide pyruvate to the TCA cycle and mitochondrial respiration for further ATP production (Zhao and Assmann, 2011; Chang et al., 2017). Glucose 6-phosphate (G6P) and phosphoenolpyruvate (PEP) can be readily converted to pyruvate (Lim et al., 2019). A previous study indicated that the overall pyruvate level is the key indicator in the control of glycolysis, entering the TCA cycle and releasing ATP to stimulate plant growth (Lim et al., 2019). In our study, glucose consumption increased with an increase in G6P, PEP, and pyruvate at stages III and IV, suggesting that the glycolysis pathway was active during germination and seedling establishment, while it was severely inhibited during maturation.

Phosphofructokinase (PFK) and pyruvate kinase (PK) are important control points in the glycolytic pathway, as they catalyze two irreversible steps (He et al., 2019). PFK regulates the level of fructose-1,6-bisphosphate, a major pathway that generates ATP from glucose (Li et al., 2013). PK catalyzes the conversion of phosphoenolpyruvate into pyruvate during glycolysis (Zeng et al., 2019). A previous study demonstrated that *OsPK1* was involved in plant morphological development, and the disruption of *OsPK5* function resulted in slow germination and seedling growth, blocked glycolytic metabolism, caused glucose accumulation, and decreased energy levels (Yang et al., 2022). In this experiment, from stage I to II, *ZmPFK* and *ZmPK* expression was downregulated, followed by a rapid increase in expression until stage IV when G6P, pyruvate, and PEP accumulated, implying the critical roles of these genes in glycolytic metabolite accumulation during *Z*. *marina* seed germination and seedling growth.

In addition, overexpression of a cytosolic glyceraldehyde-3-phosphate dehydrogenase (GAPDH) gene (*OsGAPC3*) leads to a high germination rate in rice seeds (Tan et al., 2013). Enolase (ENO) catalyzes the synthesis of 2-Phospho-D-glyceric acid (2-PGA) and PEP in the glycolytic pathway, thereby providing organic acids for seed germination (Østergaard et al., 2004; Liu et al., 2018). In our study, *ZmGAPDH*s and *ZmENO*s were actively expressed at stages III and IV, which may have accelerated glycolytic metabolism to provide 2-PGA and PEP for *Z*. *marina* seed germination and seedling growth. Notably, genes and metabolites associated with the biological processes of glycolysis were downregulated during *Z*. *marina* seed maturation and then upregulated during germination. It can be speculated that these changes may have a positive effect on seed germination and are thus repressed, maintaining a low level of metabolic activity during seed maturation to preserve seed viability and enable optimal operation.

### 4.3 TCA cycle becomes the main source of energy during *Z*. *marina* seed development

The TCA cycle is the final metabolic pathway for the decomposition of pyruvate and fatty acids, which enter as acetyl-CoA and are further oxidized to produce ATP (Liu et al., 2019; Dong et al., 2021; Gostomska-Pampuch et al., 2021). In this study, an increase in acetyl-CoA was accompanied by an increase in free fatty acids from stage II to IV, indicating that free fatty acids released from triacylglycerol are degraded to acetyl-CoA with the release of energy during *Z*. *marina* seed germination. Interestingly, although the content of pyruvate increased at stages III and IV, the pyruvate content decreased significantly compared with stage I, indicating that a large amount of pyruvate produced by glycolysis was broken down into acetyl-CoA and entered the TCA cycle. Meanwhile, acetyl-CoA entering the TCA cycle was oxidized to organic acids and produced large amounts of ATP, which provided energy for plant growth and development (Zhang and Fernie, 2018; Liu et al., 2019). In our study, the contents of organic acids (succinic acid, fumaric acid, citric acid, and isocitric acid) and ATP significantly increased at stages III and IV, indicating that the TCA cycle provides many intermediates (organic acids) and energy sources for *Z*. *marina* seed germination and seedling growth.

The expression of *MDH*, *ACO*, *FUMC*, or *PDH* encoding malate dehydrogenase, aconitase, fumarate hydratase, and pyruvate dehydrogenase, respectively, improved respiratory activity by regulating organic acid synthesis during seed germination and promoted germination (Liu et al., 2019). Our results showed that *ZmACO*s, *ZmFUMC*s, *ZmMDH*s, and *ZmPDC*s maintained a high expression level along with massive organic acid accumulation at stages III and IV, implying the critical roles of these genes in regulating the efficiency of the TCA cycle to increase energy production during seed germination.

Apart from decomposition of the glucose and fatty acids, another pathway feeding the TCA cycle is the protein catabolism (Mei et al., 2021); proteins derive from seagrasses seeds were hydrolyzed into amino acids by proteases during seed germination (Kuo et al., 1990). Acetyl CoA was not only an oxidative breakdown product of sugars and fatty acids, but also a metabolite of certain amino acids (Galili et al., 2014; Mei et al., 2021). Our study showed that the content AAs generated from protein breakdown w significantly increased from stage II to IV. Several studies have found that the added aspartic acid may enter the TCA cycle, thereby used to replenish intermediates for the TCA cycle (Vigani et al., 2016; Gao et al., 2019; Zhou et al., 2020). For example, the aspartic acid (Asp) family such as Lys, Thr, and Asn provide carbon skeletons to synthetic acetyl-CoA, which may enhance the cycle flux for the TCA cycle (Vigani et al., 2016; Su et al., 2017). In addition, catabolism of Tyr also had important effects on the synthesis of the TCA cycle metabolite fumarate succinate (He et al., 2019). In our study, the content of these amino acids was significantly increased from stage II to IV, indicating that the catabolism of amino acids provided the supply of materials to ensure efficient operation of the TCA cycle during *Z*. *marina* seed germination and seedling establishment.

### 4.4 Pentose phosphate pathway is activated during *Z*. *marina* seed germination and seedling establishment

The pentose phosphate pathway is a supplementary source of cellular energy that can directly oxidize sugars and complement glycolysis to provide the cell with NADPH and materials to promote seed germination (Wang et al., 2021). During the development of *Arabidopsis*, G6P is metabolized to erythrose 4-phosphate, ribulose 5-phosphate, and sedoheptulose 7-phosphate with the release of NADPH and ATP through the pentose phosphate pathway to promote plant development (Andriotis and Smith, 2019). Subsequently, sugar phosphate is converted to fructose 1,6-bisphosphate and then feed back into glycolysis (Wang et al., 2015; He et al., 2018). From stage II to IV, an increase in the content of erythrose 4-phosphate, ribulose 5-phosphate, and sedoheptulose 7-phosphate was accompanied by an increase in the intermediate metabolites of glycolysis, indicating that the pentose phosphate pathway was also an energy supply pathway to a certain extent during *Z*. *marina* seed germination.

Glucose 6 phosphate dehydrogenase (G6PD) and phosphogluconic acid dehydrogenase (PGD) are rate-limiting enzymes of glucose oxidative decomposition in the pentose phosphate pathway; and their activity reflects the activation state of this pathway (Yang et al., 2019). Other studies have found that an increase in G6PD, PGD, transketolase (TK), and transaldolase (TAL) activity can promote the transition of *Euscaphis konishii* seeds from dormancy to germination (Zhang et al., 2015). In our study, transcriptome analysis showed that from stage II to IV, the expression levels of *ZmG6PD*, *ZmPGD*, *ZmtktA*, and *ZmtalA* were upregulated when erythrose 4-phosphate, ribulose 5-phosphate, and sedoheptulose 7-phosphate accumulated, implying that the pentose phosphate pathway mought be activated during *Z*. *marina* seed germination.

## 5 Conclusion

In this study on the entire developmental process of *Z*. *marina* seed, we used transcriptomic and metabolomic approaches to analyze the dynamic physiological processes and molecular mechanisms, unveiling many novel findings. Starch and sucrose metabolism plays an indispensable role in maintaining the dynamic balance of sugar substances and energy homeostasis for the entire developmental process of *Z*. *marina* seed by regulating the interconversion of starch and sugar. The glycolysis pathway was inhibited at maturation but was activated and produced pyruvate to enter the TCA cycle at germination and seedling establishment. The TCA cycle was proved to be the more effective pathway to supply energy for *Z*. *marina* germination and seedling growth through an upregulation in a large number of genes and metabolites. The pentose phosphate pathway, as a metabolic bypass of glycolysis, receives its substrate from glycolysis and feeds its products back into glycolysis, thereby playing a supplementary pathway role during seed germination and seedling establishment. Collectively, the results provides new insights into changes in metabolic pathways throughout the development of *Z. marina* seeds, and provides solid evidence for large-scale restoration of seagrass beds using seed methods.

## Data availability statement

The original contributions presented in this study can be found in the article/**Supplementary Material**. The RNA‐Seq raw sequence data presented in the study are deposited in the National Center for Biotechnology Information (NCBI) Sequence Read Archive (SRA) repository, accession number SRP425505.

## Author contributions

MZ: investigation, methodology, data curation, formal analysis, software, writing – original draft. YZ: investigation, methodology, formal analysis, conceptualization, funding acquisition, resources, validation, supervision, writing – review & editing. JC: software, visualization. SS: software, visualization. SX: software, visualization. XZ: investigation, methodology. XT: data curation, conceptualization, funding acquisition, project administration, validation, supervision, writing – review & editing. All authors contributed to the article and approved the submitted version.
