# Supplementary figures and images for: Insights into the regulation of energy metabolism during the seed-to-seedling transition in marine angiosperm Zostera marina L.: Integrated metabolomic and transcriptomic analysis
Source: Front Plant Sci. 2023 Mar 10;14:1130292. doi: 10.3389/fpls.2023.1130292 (PMC10036900; doi:10.3389/fpls.2023.1130292)

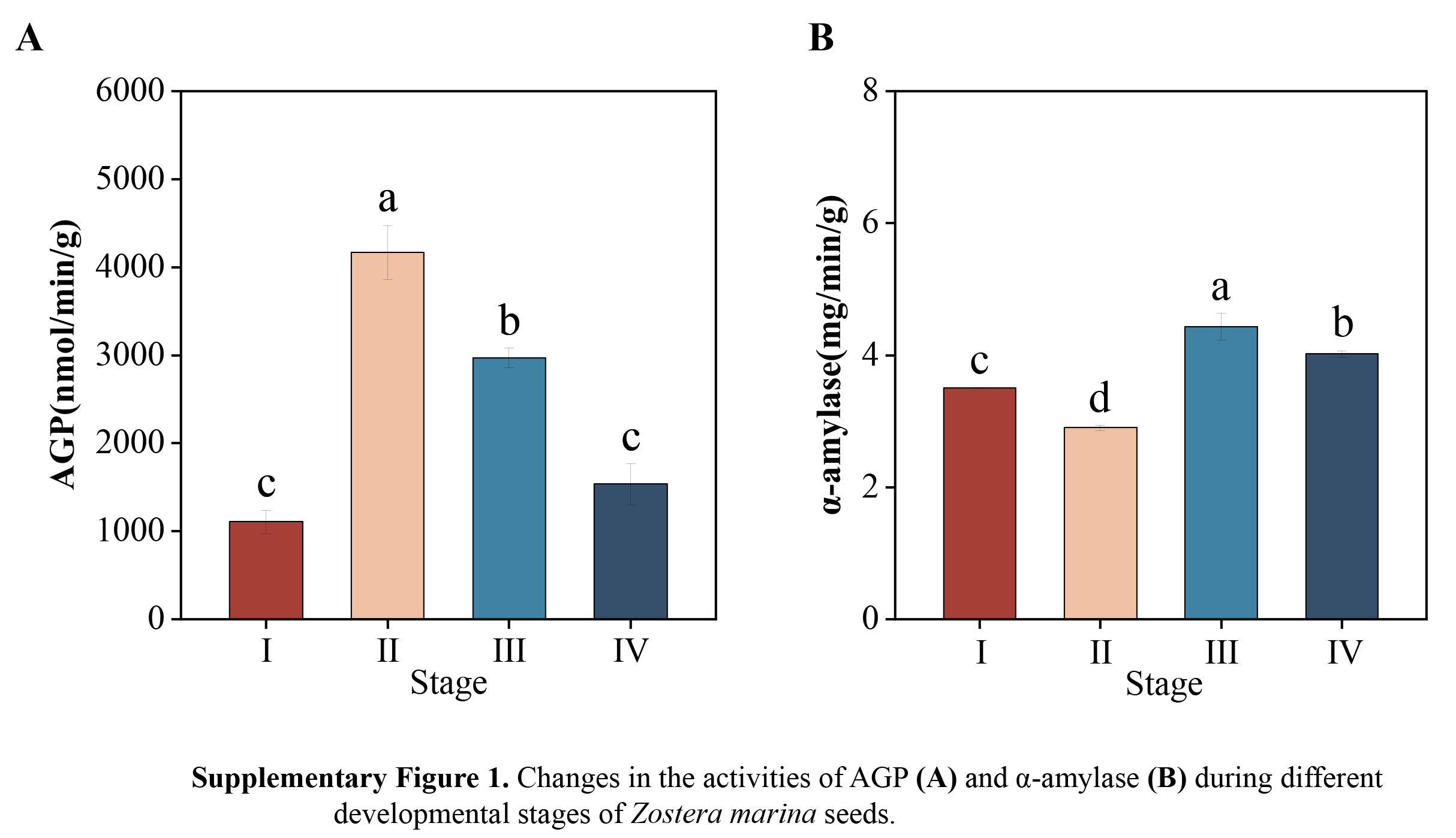

Supplement: Supplementary file 1 [file Image_1.jpeg]

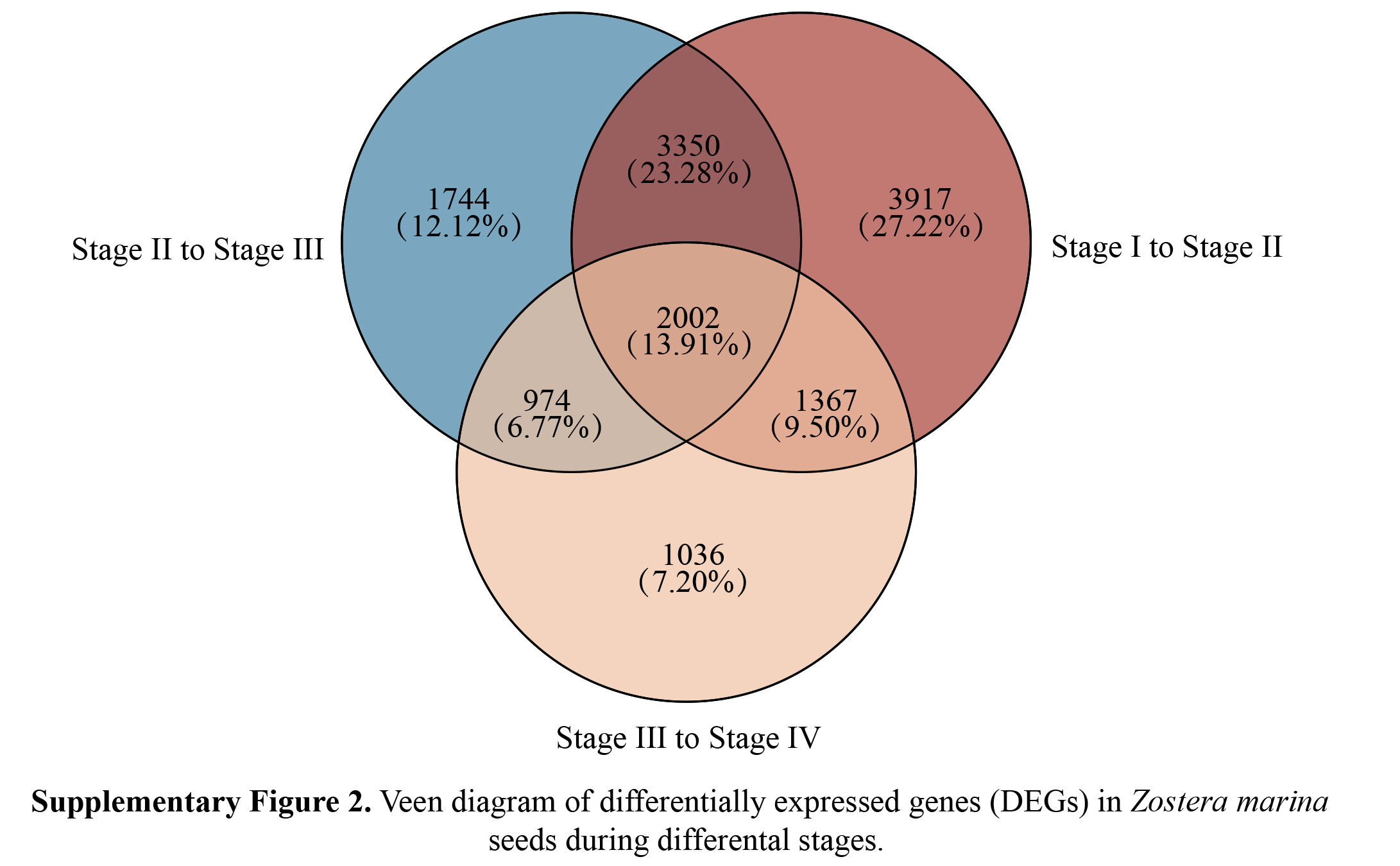

Supplement: Supplementary file 2 [file Image_2.jpeg]
